# Case Report: Ovarian dysgerminoma with pseudo-Meigs syndrome in a child

**DOI:** 10.3389/fped.2026.1773364

**Published:** 2026-02-10

**Authors:** Zhanhu Li, Dong Chen, Qiang Wei

**Affiliations:** Department of Pediatric Surgery, Children’s Hospital of Xi'an Jiaotong University, Xi'an, China

**Keywords:** ascites, children, ovarian dysgerminoma, pleural effusion, pseudo-Meigs syndrome

## Abstract

Ovarian dysgerminoma is a rare pediatric germ cell tumor, and its association with pseudo-Meigs syndrome (PMS) is exceedingly uncommon. PMS is characterized by massive pleural effusions and ascites accompanying a pelvic mass, which often leads to clinical misdiagnosis. This report describes the case of a 15-year-old female patient who presented with abdominal distension, chest tightness, and significant pleural and ascitic effusions. Following surgical resection of the tumor, the effusions resolved rapidly. Postoperative pathology confirmed the diagnosis of ovarian dysgerminoma. The patient received adjuvant chemotherapy post-surgery, recovered well, and showed no evidence of recurrence during a 9-month follow-up period.

## Introduction

Dysgerminoma is a malignant ovarian tumor that, although rare among all ovarian neoplasms, is one of the most common subtypes found in young women with malignant germ cell tumors (1). Meigs syndrome is a rare condition defined by the triad of an ovarian fibroma/thecoma, ascites, and pleural effusion (2). When the same clinical presentation occurs in association with other benign or malignant ovarian tumors (non-fibroma/non-fibroma-like tumors), it is termed pseudo-Meigs syndrome (PMS) (3). The incidence of Meigs syndrome is approximately 1% among ovarian tumors, while PMS is even rarer (4). Herein, we report a rare case of ovarian dysgerminoma complicated by PMS in a 15-year-old child in order to raise awareness about this unusual disease entity.

## Case presentation

**Figure 1:**
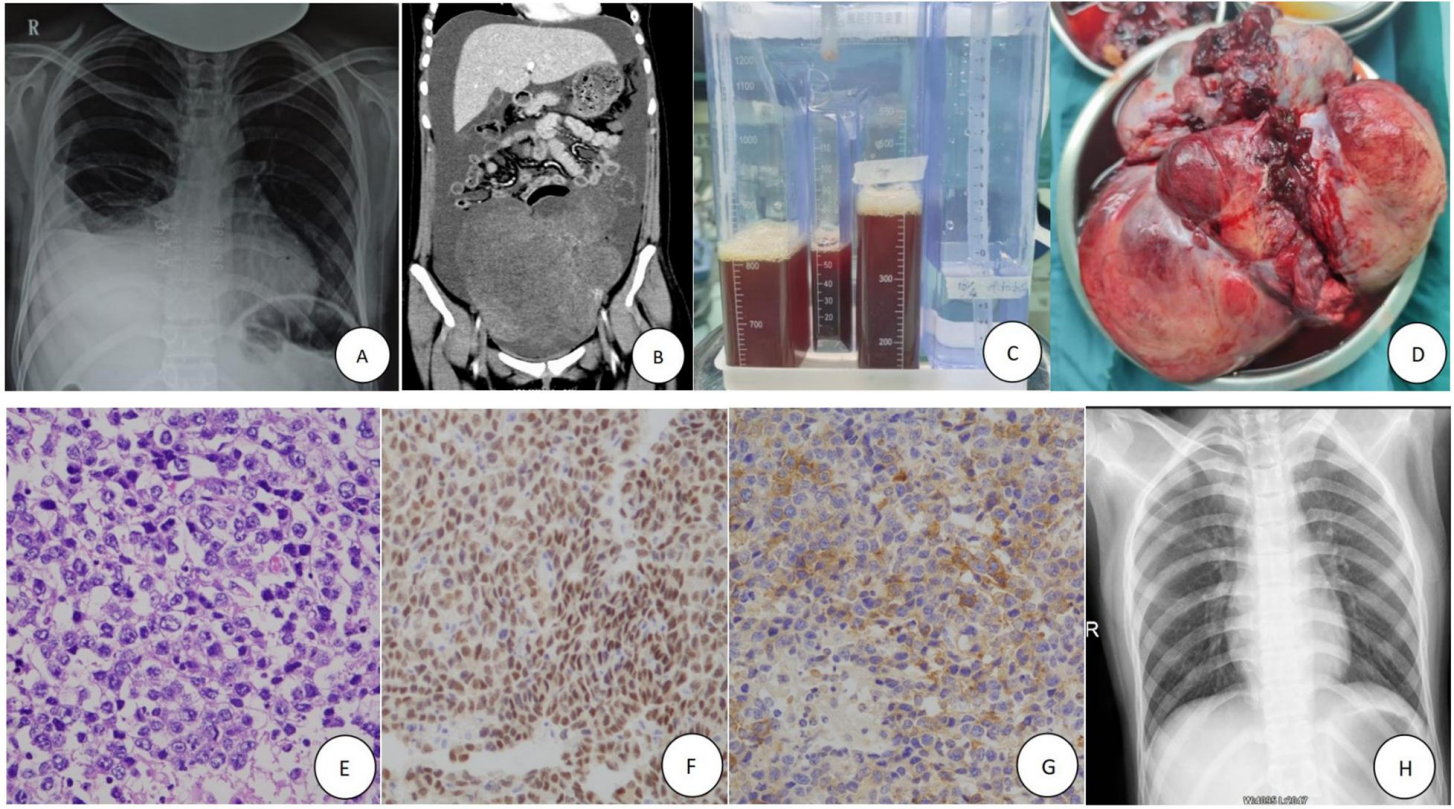
Clinical, imaging, and pathological findings of the case. **(A)** Chest x-ray on admission showing massive right pleural effusion. **(B)** Abdominal CT reveals massive ascites and a large solid tumor in the pelvis. **(C)** Closed thoracic drainage of bloody pleural fluid from the right chest. **(D)** The huge tumor (solid, irregular, partially ruptured) from the left ovary during surgery. **(E)** Hematoxylin and eosin **(H,E)** staining of the tumor tissue (×100 magnification). **(F,G)** Immunohistochemical staining indicates Otc-4+ (**F**, ×50), PLAP (focal areas, +) (**G**, ×50) in the tumor mass. **(H)** Chest x-ray on the 5th postoperative day shows resolution of the right pleural effusion.

A 15-year-old girl was admitted to our pediatric surgical ward on March 10, 2025, with a one-month history of abdominal distension, accompanied by intermittent cough and chest tightness for seven days. Physical examination of the abdomen and pelvis revealed a large, well-delineated, solid mass palpable extending to 5 cm above the umbilicus. Chest x-ray demonstrated massive right pleural effusion (Figure 1A), and thorax computed tomography (CT) confirmed the effusion with no evidence of metastases. Contrast-enhanced abdominopelvic CT scan showed a 20cm × 18cm × 15 cm solid mass in the left adnexa and significant ascites (Figure 1B).

Blood tests revealed elevated C-reactive protein (53.71 mg/L) and lactate dehydrogenase (LDH, 490 U/L). Serum tumor markers were as follows: alpha-fetoprotein 2.0 ng/mL (normal: 0–10 ng/mL), carbohydrate antigen 125 (CA125) 1,210 U/mL (normal: 0–35 U/mL), beta-human chorionic gonadotropin (β-hCG) 2,126 mIU/mL (normal: 0–3 mIU/mL). Estradiol, follicle-stimulating hormone, and luteinizing hormone levels were within normal ranges. Thoracentesis was performed for symptomatic relief, draining a large volume of light bloody fluid (Figure 1C). Cytological examination was negative for malignant cells. The pleural effusion was classified as an exudate based on Light's criteria (pleural fluid/serum protein ratio >0.5 and pleural fluid/serum LDH ratio >0.6). Based on these findings, a diagnosis of an ovarian tumor with suspected Meigs or PMS was made.

After completing preoperative evaluations, the patient underwent exploratory laparotomy. Approximately 5,000 mL of light bloody ascites was aspirated intraoperatively. Exploration revealed a tumor originating from the left ovary. The tumor was irregular, solid, and partially ruptured, measuring approximately 21 cm × 19 cm × 14 cm (Figure 1D). The right ovary, fallopian tubes, peritoneum, greater omentum, mesentery, intestines, liver, and pelvic cavity showed no abnormalities or signs of tumor dissemination. Mesenteric lymph nodes were uninvolved.

Histopathological examination revealed a solid tumor with gray-red to gray-yellow cut surfaces, a fish-flesh appearance, and a soft consistency with extensive hemorrhagic necrosis. Tumor cells displayed clear to eosinophilic cytoplasm and large nuclei with vesicular chromatin and prominent nucleoli. Focal papillary architectural patterns were observed, with areas showing coarser chromatin and increased nuclear-to-cytoplasmic ratios. Mitotic figures are readily identifiable (Figure 1E). Confluent areas of hemorrhage and necrosis permeated the tumor parenchyma. There was no evidence of metastasis to the left fallopian tube. Immunohistochemical staining results were as follows: Calretinin (-), CD30 (-), CD117 (-), CK20 (-), CK7 (-), D2-40 (+), Otc-4 (+), PLAP (focal areas +), Inhibin-α(-), α-fetoprotein (-) (Figures 1F,G). The final pathological diagnosis was a giant germ cell tumor, consistent with dysgerminoma in both morphology and immunophenotype.

**Figure 2:**
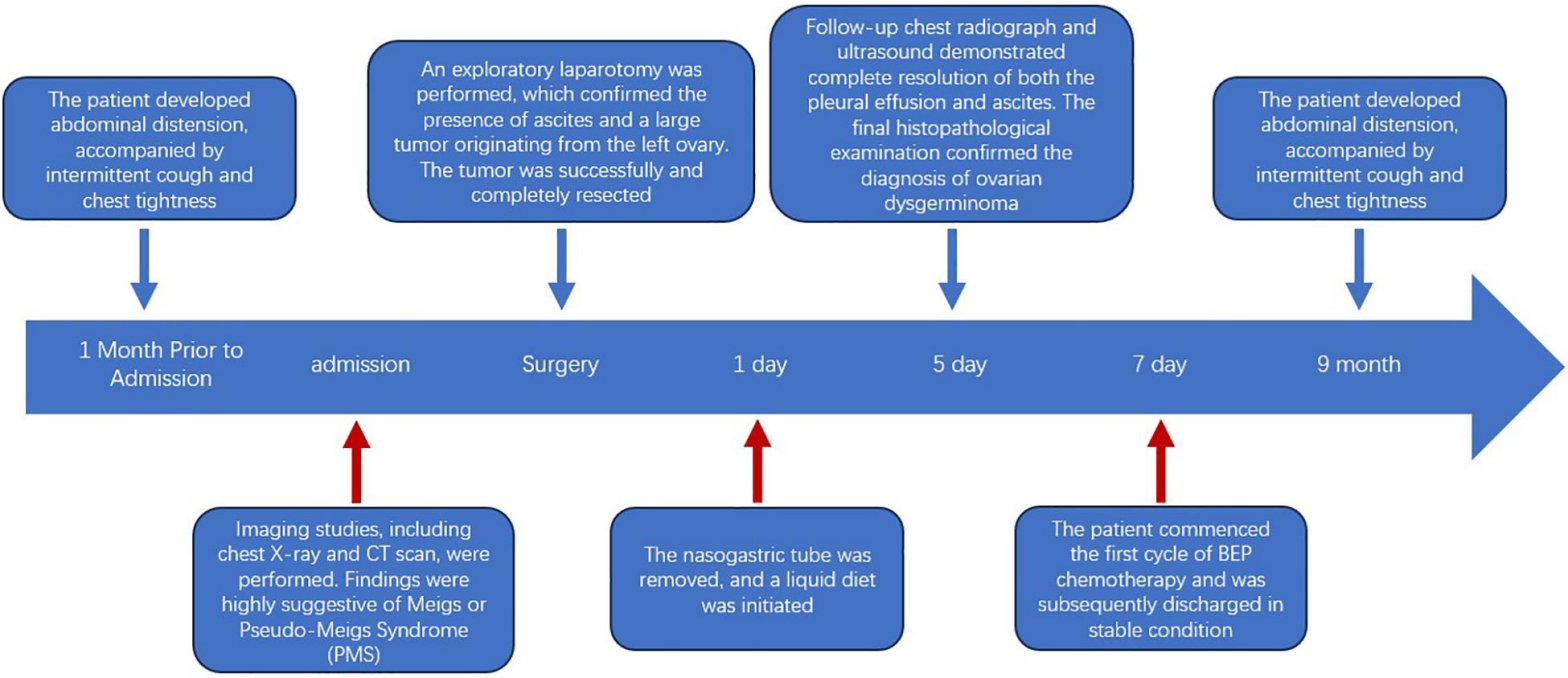
Complete timeline, including diagnosis, surgery, postoperative recovery and follow-up.

The postoperative course was uneventful, Chest radiograph on postoperative day 5 showed complete resolution of the pleural effusion (Figure 1H). Positron emission tomography-computed tomography (PET-CT) revealed no signs of lung metastasis. Given the tumor's large size and capsular rupture, it was classified as International Federation of Gynecology and Obstetrics (FIGO) stage II. 2161. The patient received BEP chemotherapy (bleomycin, etoposide, cisplatin). Serial tumor marker monitoring showed decreased serum levels at two weeks (NSE 12 ng/mL, CA125 46.58 U/mL, β-hCG 11.50 mIU/mL), with normalization of all parameters within one month. Surveillance for recurrence was performed every three months, and no recurrence was observed during the 9-month follow-up period. We used a timeline to visually display the entire treatment process (Figure 2).

## Discussion

Ovarian dysgerminoma, though the most common malignant germ cell tumor of the ovary in young women, remains a rare entity in the pediatric population (1). Its presentation with PMS, characterized by a pelvic mass, ascites, and pleural effusion, constitutes an exceedingly rare clinical scenario, particularly in children (5). This case underscores the diagnostic challenges and highlights key management principles for this unusual combination.

The diagnosis of PMS in this case was primarily one of exclusion and correlation. The triad of a large pelvic mass, ascites, and pleural effusion initially raised strong suspicion for advanced malignant disease with peritoneal and pleural metastases. Key differential diagnoses considered included: 1) Malignant pleural metastasis, which was ruled out by the absence of malignant cells on cytological examination of the pleural fluid and the lack of pulmonary or pleural lesions on CT and subsequent PET-CT; 2) Tuberculous pleurisy and peritonitis, which was considered unlikely in the absence of systemic symptoms (*e.g.*, fever, night sweats), negative fluid cytology for acid-fast bacilli, and the rapid resolution of effusions post-tumor resection without anti-tuberculous therapy; and 3) Other causes of transudative effusions (*e.g.*, cardiac, hepatic, renal), which were inconsistent with the patient's clinical profile and the exudative nature of the pleural fluid as per Light's criteria. The definitive diagnosis of PMS was established by the rapid and complete resolution of both ascites and pleural effusion following the removal of the ovarian dysgerminoma, fulfilling the core diagnostic criterion for this syndrome.

In PMS, fluid accumulation resolves following resection of the ovarian tumor (6). The pathophysiology of PMS remains unclear. Ascites is presumed to result from peritoneal irritation, inflammation, and venous/lymphatic obstruction by the tumor. Pleural effusion is thought to occur due to the translocation of ascites via diaphragmatic pores or lymphatics (7, 8). In this case, the pleural effusion was an exudate. It has been speculated that inflammatory or vasoactive mediators released by the tumor or adjacent irritated tissues might increase peritoneal capillary permeability, contributing to exudative ascites formation, which then transdiaphragmatically migrates to the pleural space. Some studies have found that the composition of ascites and pleural effusion is similar. Furthermore, when the tumor has not been removed, pleural effusion can rapidly re-accumulate after being drained via thoracentesis, which supports this hypothesis (9). However, this remains a hypothesis, and our case does not provide specific evidence to confirm this mechanism.

This represents the second reported case of PMS caused by dysgerminoma and the first pediatric case of PMS associated with elevated CA125 and β-hCG levels due to dysgerminoma (5). An ovarian mass, ascites, pleural effusion, and elevated CA125 often suggest the presence of malignant disease and/or metastasis. Immunohistochemical studies have demonstrated that elevated serum CA125 levels in patients with Meigs syndrome primarily result from increased expression of CA125 in the peritoneal and omental mesothelium, rather than from the tumor itself, and the volume of ascites positively correlates with the rise in CA125 levels (10). Although dysgerminoma does not consistently affect hormone secretion, positive HCG expression in syncytiotrophoblast giant cells has been observed in 5% of cases. In our patient, histopathological findings were diagnostic of dysgerminoma, and the absence of malignant cells in the pleural fluid ruled out pleural metastasis.

The cornerstone of treating PMS caused by ovarian dysgerminoma is resection of the primary tumor. As dysgerminoma is malignant, open abdominal surgery is recommended to ensure complete excision and minimize the risk of intraoperative rupture and dissemination. The prognosis is excellent, with tumor resection alone often sufficient for localized disease. Platinum-based chemotherapy is highly effective for locoregionally advanced disease, with a 5-year overall survival rate exceeding 90% (11). The BEP regimen is internationally recognized as first-line standard chemotherapy for ovarian malignant germ cell tumors (12). Chemotherapy for pediatric and adolescent patients should be individualized based on age, surgical extent, histology, and disease stage (13). For PMS due to ovarian dysgerminoma, stage Ia disease may be managed with surgery alone, while higher stages or high-risk factors (*e.g.*, tumor rupture) warrant adjuvant chemotherapy (6, 8). The BEP regimen is effective and typically does not compromise ovarian hormonal function (14). In our case, intraoperative tumor rupture resulted in a FIGO stage II classification (intermediate-risk). The patient received three cycles of postoperative BEP chemotherapy, recovered well, and showed no recurrence during a 9-month follow-up period.

This report has certain limitations. It is a single-center case description, and the rarity of PMS associated with dysgerminoma means our findings require validation through the accumulation of more cases in multi-center studies or registries. Furthermore, the exact pathophysiological mechanism linking dysgerminoma to massive effusions remains speculative and warrants further investigation. Despite these limitations, this case carries important clinical implications. It highlights that in adolescents and young women presenting with the triad of a pelvic mass, ascites, and pleural effusion, PMS should be considered in the differential diagnosis, even in the presence of significantly elevated tumor markers such as CA125 and β-hCG. Recognizing this entity is crucial to avoid misdiagnosing a potentially curable condition (localized tumor with paraneoplastic effusions) as an advanced, metastatic malignancy, thereby guiding appropriate surgical intervention and avoiding unnecessary pessimism regarding prognosis.

## Conclusions

We present a rare pediatric case of ovarian dysgerminoma associated with PMS. This diagnosis should be considered in young patients presenting with a pelvic mass, ascites, and pleural effusion. Although rare in children, the prognosis for PMS caused by ovarian dysgerminoma is favorable. Effusions typically resolve rapidly after tumor resection, and the decision for adjuvant chemotherapy should be guided by tumor staging.

## Data Availability

The raw data supporting the conclusions of this article will be made available by the authors, without undue reservation.
